# Transcriptional response of lignin-degrading enzymes to 17α-ethinyloestradiol in two white rots

**DOI:** 10.1111/1751-7915.12007

**Published:** 2012-11-22

**Authors:** L Přenosilová, Z Křesinová, A Slavíková Amemori, T Cajthaml, K Svobodová

**Affiliations:** 1 Laboratory of Environmental Biotechnology, Institute of Microbiology ASCR,v.v.i.Videnska 1083, 14220, Prague, Czech Republic; 2 Department of Biochemistry, Faculty of Science, Charles University in PragueHlavova 8, 12840, Prague 2, Czech Republic; 3 Institute of Environmental Studies, Faculty of Science, Charles UniversityBenatska 2, 128 01, Prague 2, Czech Republic

## Abstract

Fungal, ligninolytic enzymes have attracted a great attention for their bioremediation capabilities. A deficient knowledge of regulation of enzyme production, however, hinders the use of ligninolytic fungi in bioremediation applications. In this work, a transcriptional analyses of laccase and manganese peroxidase (MnP) production by two white rots was combined with determination of pI of the enzymes and the evaluation of 17α-ethinyloestradiol (EE2) degradation to study regulation mechanisms used by fungi during EE2 degradation. In the cultures of *Trametes versicolor* the addition of EE2 caused an increase in laccase activity with a maximum of 34.2 ± 6.7 U g^−1^ of dry mycelia that was observed after 2 days of cultivation. It corresponded to a 4.9 times higher transcription levels of a laccase-encoding gene (*lacB*) that were detected in the cultures at the same time. Simultaneously, pI values of the fungal laccases were altered in response to the EE2 treatment. Like *T. versicolor*, *Irpex lacteus* was also able to remove 10 mg l^−1^ EE2 within 3 days of cultivation. While an increase to *I. lacteus* MnP activity and MnP gene transcription levels was observed at the later phase of the cultivation. It suggests another metabolic role of MnP but EE2 degradation.

## Introduction

17α-ethinyloestradiol (EE2), a synthetic oestrogen, belongs to the group of chemicals so-called endocrine disruptive compounds (EDCs) that disrupt endocrine system in wild life. It contaminated the environment via wastewaters. It was identified as one of major oestrogenic EDCs in sewage treatment effluents (Desbrow *et al*., 1998).

Like other organic pollutants, EE2 was shown to be biodegradable by various microorganisms and their enzymes (Asgher *et al*., 2008; Cajthaml *et al*., 2009a). In white rot fungi, EE2 degradation has been often connected with ligninolytic enzyme activities (Suzuki *et al*., 2003; Blanquez and Guieysse, 2008; Cajthaml *et al*., 2009a). Cajthaml and colleagues (2009b) tested eight fungal strains for EE2 removal in a complex culture medium. The highest EE2 removal efficiency was observed with *Irpex lacteus*, *Pleurotus ostreatus* and *Pycnoporus cinnabarinus*. Similarly to other works (Cheong *et al*., 2006; Šušla and Svobodová, 2008; Takamiya *et al*., 2008; Yeo *et al*., 2008), alterations in the enzyme activity levels in the fungal cultures were observed during the EE2 degradation.

Enzyme production in fungi can be regulated at different levels by the pollutants. Saparrat and colleagues (2010) demonstrated a differential regulation of laccase genes in *Coriolopsis rigida*. The gene *lcc1* was then shown to code for laccases involved in the degradation of olive mill wastewaters (Díaz *et al*., 2010). Laccase expression in fungi is known to be regulated at the level of gene transcription by various aromatic compounds and metal ions. The role of gene-promotor regions in the process has been reviewed in Piscitelli and colleagues (2011). Fujihiro and colleagues (2009) described that *Trametes versicolor* UAMH 8272 produced two groups of laccases, each of which included several isoforms showing different pI values. The enzymes differed in their capability of PCB degradation. A possible relationship of *T. versicolor* laccase pI form and its PCB degradation potential was also suggested in our previous work (Plačková *et al*., 2012).

This article presents a more complex study of the changes in the laccase and MnP production caused by the addition of EE2 to the cultures of *Trametes versicolor* CCBAS 612 and *Irpex lacteus* CCBAS 931 (fungal strains were obtained from the culture collection CCBAS, Institute of Microbiology ASCR,v.v.i., Czech Republic), two representatives of white rot fungi capable of EE2 degradation. Transcriptional levels of genes encoding laccase and MnP were correlated with time profiles of the enzyme pI forms and EE2 degradation by fungal cultures and supernatants. The results showed that alterations in enzyme transcription levels do not necessarily correlate with changes in enzyme pI values and EE2 degradation capacity of fungal cultures. Two studied fungal strains differed in the reactions evoked by EE2.

## Results and discussion

### Enzyme activities in liquid cultures

**Figure 1:**
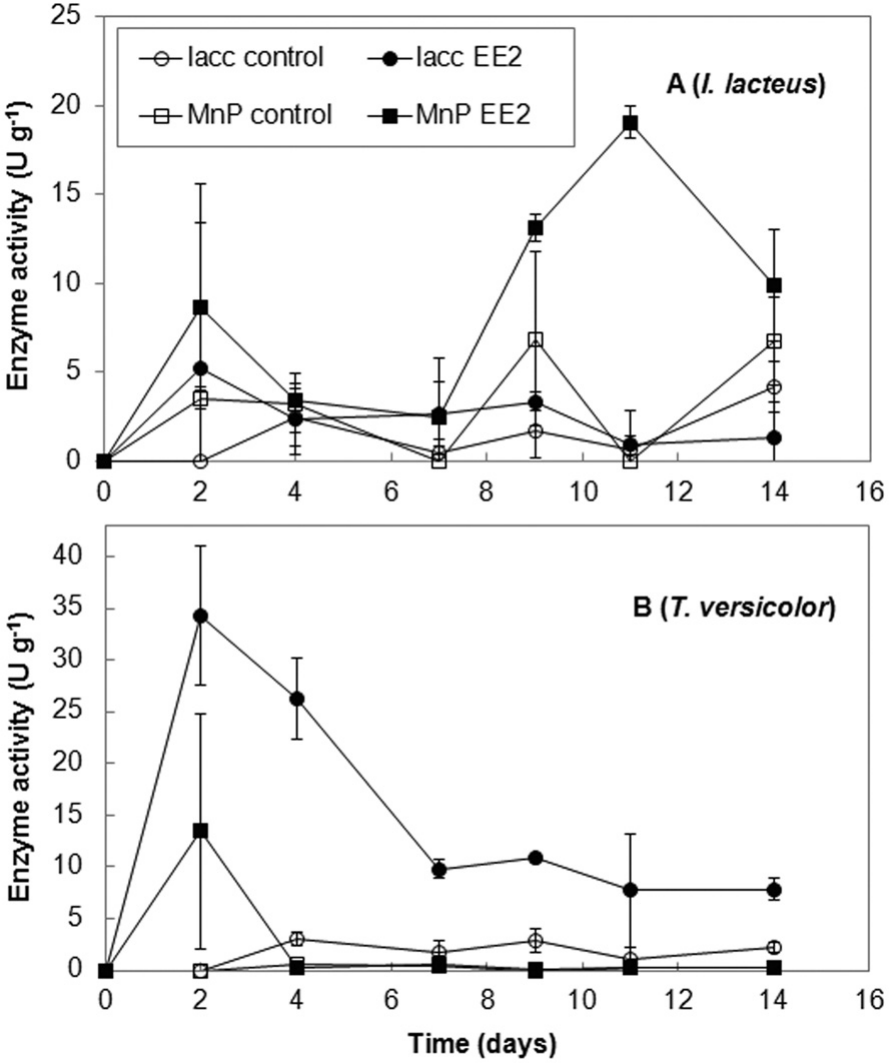
Enzyme activities detected in the cultures of *I. lacteus* (A) and *T. versicolor* (B). Laccase activities were measured by the oxidation of ABTS (Matsumura *et al*., 1986). Manganese peroxidase was assayed with 2,6-dimethoxyphenol (DeJong *et al*., 1994). Enzyme activities were expressed in units of the enzyme activity (U; 1 unit oxidized 1 μmol of substrate per minute) per gram of a dry weight of fungal biomass present in the cultures. Laccase (circles), manganese peroxidase (squares), EE2-stimulated cultures (full symbols), control cultures (open symbols).

*T. versicolor* and *I. lacteus* were cultivated in 20 ml of a mineral liquid medium (MM) according to Tien and Kirk (1988) that was supplemented with 10 mg l^−1^ EE2. In both fungi, the addition of EE2 to fungal cultures had no effect on the fungal growth. The cultures reached a maximum of biomass of 138 ± 18 mg of dry weight (*T. versicolor*) and 51 ± 3 mg of dry weight (*I. lacteus*) after 14 days of cultivation. It shows that both *T. versicolor* and *I. lacteus* can resist water stress induced by EE2 via chaotropicity-mediated mechanism (Bhaganna *et al*., 2010). Laccase and MnP activities were detected in all fungal cultures and their values were normalized to fungal biomass (Fig. 1A and B). Both fungi were able to degrade the whole amount of EE2 (0.2 mg) within 3 days of cultivation.

A biodegradation potential of white rot fungi has been often linked to their ligninolytic enzyme apparatus. Some of their characteristics give the enzymes an advantage in biotechnological applications as summarized in Ruiz-Duenas and Martinez (2009). In several biodegradation studies, significant changes in the enzyme expression have been observed during the biodegradation processes (Cheong *et al*., 2006; Šušla and Svobodová, 2008; Takamiya *et al*., 2008; Yeo *et al*., 2008; Yang *et al*., 2006). However, the connection between the altered enzyme expression and biodegradation was not clear in some of the recent works (Sole *et al*., 2008; Plačková *et al*., 2012). In this work, changes in the production of *I. lacteus* MnP and *T. versicolor* laccase were observed in the cultures during the degradation of EE2.

The addition of EE2 to *I. lacteus* cultures significantly enhanced the MnP activity (Fig. 1A). The MnP production reached its maximum of 19 ± 0.9 U g^−1^ of dry mycelia after 11 days and was 2.7 times higher than MnP activity maximum of untreated control cultures (6.9 ± 4.9 U g^−1^, reached after 9 days of cultivation). Laccase activity was low in *I. lacteus* cultures and it was unaffected by the addition of EE2.

In *T. versicolor* cultures, EE2 caused an increase in laccase activity with the effect being most prominent at the beginning of the cultivation (Fig. 1B). In the EE2-spiked cultures of *T. versicolor*, laccase activity maximum amounted 34.2 ± 6.7 U g^−1^ of dry mycelia after 2 days of cultivation. Compared with that, laccase activity in the control cultures did not exceed 3 U g^−1^ of dry mycelia.

### Laccase gene expression in *T. versicolor*

**Figure 2:**
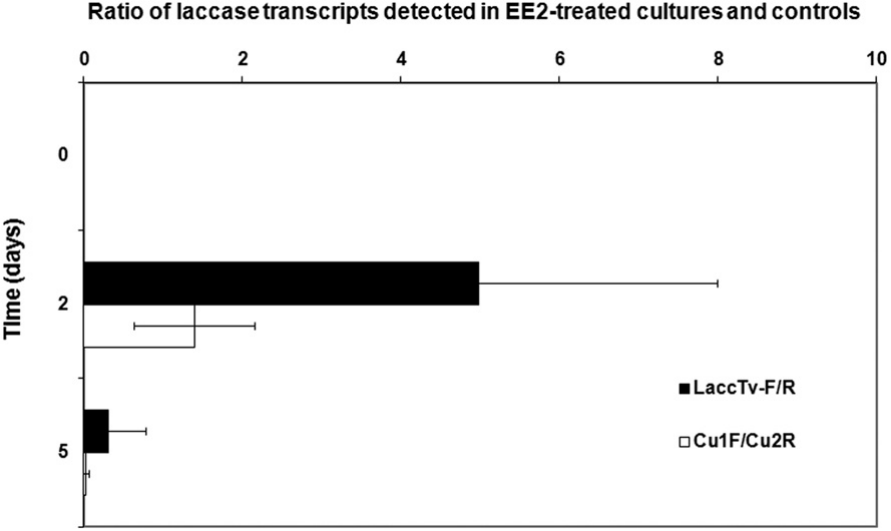
Laccase transcript levels detected in *T. versicolor* cultures by qPCR. Degenerated Cu1F and Cu2R primers (Luis *et al*., 2004) and *T. versicolor*-specific LaccTv-F and LaccTv-R primers (Hiscox *et al*., 2010) were used for laccase gene detection. A standard two-gene quantification strategy with PCR efficiency correction was then used and gene transcript levels were expressed as a ratio of the transcript levels detected in EE2-treated and untreated control cultures (Plačková *et al*., 2012).

Using degenerated Cu1F and Cu2R primers, laccase gene fragments (*c*. 200 bp) were amplified from cDNA samples obtained from *T. versicolor* cultures. Real-time PCR results showed a comparable laccase mRNA synthesis in EE2-spiked cultures and untreated cultures 2 days after the addition of EE2 (Fig. 2). After 5 days of cultivation, laccase gene expression dramatically decreased in EE2-treated cultures compared with the control cultures. With specific LaccTv-F and LaccTv-R primers, a ratio of laccase transcript levels of 4.9 ± 3 was observed in EE2-treated cultures compared with the control cultures on day 2 of cultivation when analysed (Fig. 2). In the later phase (5 days after the addition of EE2), the ratio decreased to a value of 0.31 ± 0.48 indicating that the laccase gene expression was lower in the EE2-spiked cultures compared with the control ones.

The laccase gene segment amplified from *T. versicolor* gDNA with LaccTv-F and LaccTv-R primers was gel cleaned and sequenced with the forward LaccTv-F primer. blastn analyses of the obtained sequence (492 bp, NCBI database Accession No. JX050201) showed 86% identity of the sequence with the *Trametes* sp. AH28-2 *lacB* gene (NCBI database Accession No. AY846842.1).

The transcript levels of the laccase gene (Fig. 2) detected using LaccTv-F and LaccTv-R primers, positively correlated with the increase in laccase activity in *T. versicolor* cultures (Fig. 1B). On the other hand, laccase expression analyses that was conducted with the degenerated Cu1F and Cu2R primers showed that the overall laccase transcript levels detected in the EE2-spiked cultures on the day 2 of cultivation were comparable with those of control cultures (Fig. 2). It indicates that the expression of an unidentified laccase gene was probably lowered after the treatment with EE2 to compensate for the increase in the transcription of *lacB* gene. A differential expression of laccase genes in *Trametes* sp. AH28-2 was also described by Xiao and colleagues () and the *lacB* gene was shown to be selectively induced by 3,5-dihydroxytoluene. In contrast to that, *lacA* gene from *Trametes* sp. AH28-2 was shown to play a role in biomass saccharification (Zhang *et al*., 2012). Laccase gene *lac48424-1* from *Trametes* sp. 48424 was shown to code for a laccase with a strong ability for decolorizing synthetic dyes (Fan *et al*., 2011). After 5 days of cultivation *lacB* transcription in the EE2-spiked cultures of *T. versicolor* dropped bellow the transcription levels in the control cultures indicating that the laccase activity remaining in the culture supernatants was more likely a result of enzyme stability than *de novo* synthesis. Higher expression levels of *T. versicolor* laccases were also observed during degradation of 2,4,6-trinitrotoluene (Cheong *et al*., 2006) and bisphenol A (Takamiya *et al*., 2008) which suggests the enzyme to be a part of the general organopollutant related stress response of the fungus. However, generalization of the results for other fungal species is not possible. A transcriptional profiling of *P. ostreatus* laccase genes showed that laccase gene family transcription profiles can greatly differ even between closely related strains (Castanera *et al*., 2012).

### MnP gene expression in *I. lacteus*

**Figure 3:**
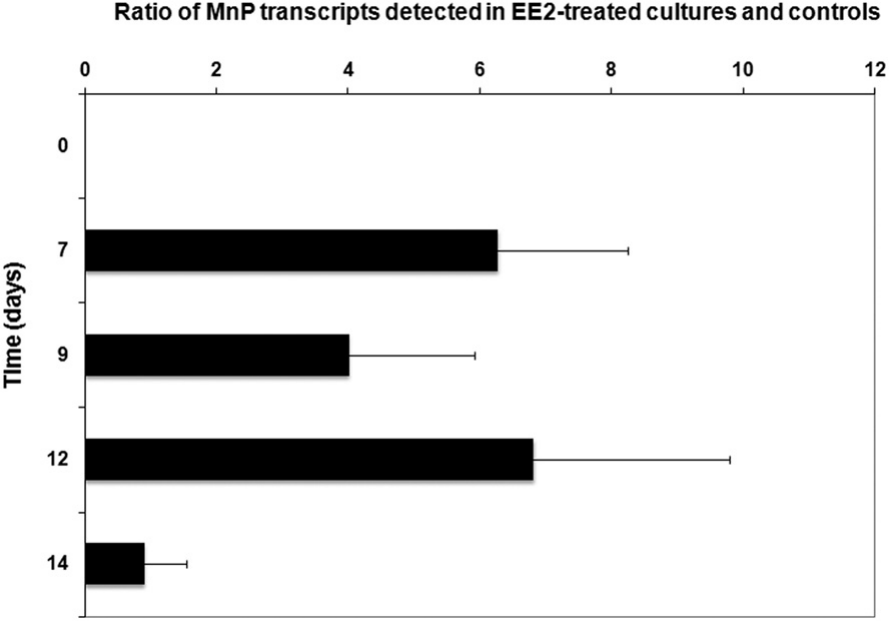
MnP transcript levels detected in *I. lacteus* cultures by qPCR using the degenerated mnp-U and mnp-L primers (Nagai *et al*., 2007). A standard two-gene quantification strategy with PCR efficiency correction was then used and gene transcript levels were expressed as a ratio of the transcript levels detected in EE2-treated and untreated control cultures (Plačková *et al*., 2012).

In the *I. lacteus* cultures, the expression of MnP genes was analysed using the degenerated mnp-U and mnp-L primers. In this case, gene fragments of *c*. 500 bp were amplified from cDNA samples. Real-time PCR analyses of MnP transcript levels showed 4–6.8 times higher MnP expression in the EE2-treated cultures compared with the control ones 7–12 days after the addition of EE2 to the cultures (Fig. 3). After that, MnP expression in the EE2-treated cultures decreased to the level that was detected in the control cultures. The results showed that the MnP activity in the culture supernatants (Fig. 1A) corresponded well to the levels of MnP transcripts detected in the cultures. The expression of analysed genes was then most probably responsible for the enzyme activities present in the culture liquids.

On the other side, the fungal cultures of *I. lacteus* were able to remove 10 mg l^−1^ EE2 completely within the first 3 days of cultivation. The stimulation of MnP production occurred after the EE2 degradation. The enzymes thus could not play a role in the initial attack of EE2. However, they could participate in the later degradation of EE2 intermediates or other fungal processes. Berne and colleagues (2008) showed that another hormone progesterone induced a global adaptive stress response in the filamentous fungus *Cochliobolus lunatus* including changes in genes involved in vesicle mediated transport, derivative metabolism, lipid metabolism or cell wall biogenesis. In *Paracoccidioides* an effect of 17 beta-oestradiol on the regulation of cell dimorphism was characterized (Shankar *et al*., 2011). On the other hand, EE2 metabolites produced by *I. lacteus* cultures could also participate in the induction of MnP transcription.

### IEF analyses of culture supernatants

Supernatants from *T. versicolor* and *I. lacteus* cultures grown in the presence/absence of EE2 were analysed for the presence of laccase and MnP forms, respective. Aliquots corresponding to 0.12–1.13 mU of MnP activity and 0.15–0.93 mU of laccase activity were subjected to a native IEF. The pI values of detected enzyme forms are summarized in Table 1. In *I. lacteus* cultures, two MnP forms were found except the 12-day-old EE2-spiked cultures. The pI values of the enzymes varied in the range of 3.98–4.43. In *T. versicolor*, the addition of EE2 to the cultures led to the production of three laccase forms of lower pI values compared with a laccase form that was detected in EE2-unspiked control culture.

**Table 1:** pI of *I. lacteus* MnP and *T. versicolor* laccase detected in the supernatants of fungal cultures using a native IEF analyses

Time of cultivation (days)	pI values of fungal enzymes	
	Control cultures	EE2-stimulated cultures
*I. lacteus* MnP		
7	4.13; 4.20	3.98; 4.05
9	4.05; 4.13	4.28; 4.31
12	4.35; 4.43	4.31
14	4.20; 4.28	4.17; 4.24
*T. versicolor* laccase		
1	4.05; 4.13	4.05; 4.13
2	4.05	4.05
5	4.17	3.93; 4.01; 4.09

The IEF gel (7.5% w/v) was prepared using ampholines of pI 2.5–5.0 and 3.5–10.0 (Pharmacia). Gels were activity-stained with 5 mM ABTS (laccase) and 2 mM 2,6-dimethoxyphenol in the presence of 1 mM MnSO_4_ and 0.5 mM H_2_O_2_ (MnP).

In addition to the enzyme activity and gene expression levels, the composition of pI forms of the enzymes was thus also altered in the fungal cultures in response to the addition of EE2 showing that the enzyme production was regulated at more than one level. Similarly to PCB compounds (Plačková *et al*., 2012), the addition of EE2 to the *T. versicolor* cultures at the beginning of the cultivation led to the production of a higher number of laccase forms compared with the control cultures with pI values of EE2-induced enzymes being slightly higher than those of PCB-induced ones (Plačková *et al*., 2012). *I. lacteus* produced mostly two forms of MnP in both EE2-spiked and control cultures. The pI profile of the cultures only slightly varied in response to EE2 and during the cultivation. The pI values of MnPs were lower than those detected in the immobilized cultures of *I. lacteus* after the treatment with synthetic dyes and less enzyme forms were produced (Šušla and Svobodová, 2008).

### *In vitro* degradation of EE2

**Figure 4:**
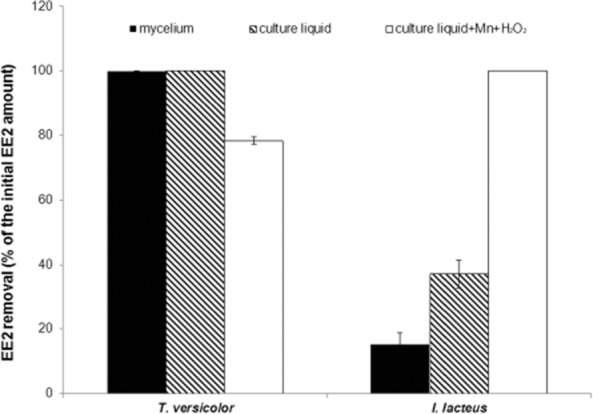
*In vitro* degradation of EE2 by fungal mycelium and culture supernatants. 10 mg l^−1^ EE2 was incubated with a sample of homogenized fungal mycelium and culture liquid for 24 h at 28°C. Reaction mixtures contained 60 mM NaAc buffer (pH 5.0), 10 mg l^−1^ EE2, 2.5% (v/v) DMSO, and 0.4 ml of culture liquid/60 mg of homogenized mycelium in a final volume of 2 ml. A parallel set of samples were incubated in the presence of 1 mM MnSO_4_ and 0.5 mM H_2_O_2_. Culture liquids and mycelium inactivated by 100°C for 30 min served as controls. After the incubation samples were extracted with ethyl acetate and subjected to HPLC analyses.

Culture supernatants and mycelium from 10-day-old EE2-spiked fungal cultures were tested for their ability to degrade 10 mg l^−1^ EE2 *in vitro*. In case of *T. versicolor*, 100% of EE2 was removed by mycelial samples and by the aliquots of the concentrated culture liquid within 24 h (Fig. 4). The samples of culture liquid contained 175 mU of laccase activity. In the mycelial samples 10 mU of laccase activity were detected. The addition of Mn ions and hydrogen peroxide to the culture liquid resulted in a partial inhibition of EE2 removal. An evidence for the implication of laccases in EE2 degradation by *T. versicolor* was brought already in the previous work of Blanquez and Guieysse (2008) and EE2 degradation by pure or partially purified laccases from *T. versicolor* has also been reported (Suzuki *et al*., 2003; Auriol *et al*., 2007). The results of EE2 incubation *in vitro* with *T. versicolor* mycelium showed that mycelium-associated enzyme activities could also participate on the EE2 degradation by *T. versicolor* similarly to the decolorization of Amaranth that was described previously (Swamy and Ramsay, 1999). To summarize, laccase that is expressed by *T. versicolor* in response to EE2 cooperates in EE2 degradation most likely with mycelium-associated enzymes.

Samples of *I. lacteus* culture liquid were able to remove 37.1 ± 4.3% of EE2 within 24 h. Whereas in the presence of Mn ions and hydrogen peroxide, 100% of EE2 was removed within the same time (Fig. 4). The samples of culture liquids contained 1.8 mU of laccase activity and 26.1 mU of MnP. The lowest EE2 degradation was observed with the samples of *I. lacteus* mycelium that showed laccase activity of 0.6 mU. The results indicated that MnP activities, which were produced by the fungus in response to the EE2 treatment, possessed an ability to degrade EE2. However, for the belated set up of their production, the MnP activities could not participate in the EE2 degradation observed in the fungal cultures.

## Conflict of interest

None declared.
